# Foliar δ^13^C response patterns along a moisture gradient arising from genetic variation and phenotypic plasticity in grassland species of Inner Mongolia

**DOI:** 10.1002/ece3.453

**Published:** 2012-12-27

**Authors:** Yanjie Liu, Haishan Niu, Xingliang Xu

**Affiliations:** 1 College of Resources and Environment, University of Chinese Academy of SciencesBeijing, 100049, China; 2 Key Laboratory and Ecosystem Network Observation and Modelling, Institute of Geographic Sciences and Natural Resources Research, Chinese Academy of SciencesBeijing, 100101, China

**Keywords:** Genetic, *Leymus chinensis*, plastic, *Stipa grandis*, WUE, δ^13^C

## Abstract

Plants depend upon both genetic differences and phenotypic plasticity to cope with environmental variation over different timescales. The spatial variation in foliar δ^13^C levels along a moisture gradient represents an overlay of genetic and plastic responses. We hypothesized that such a spatial variation would be more obvious than the variation arising purely from a plastic response to moisture change. *Leymus chinensis* and *Stipa* spp. were sampled from Inner Mongolia along a dry-wet transect, and some of these species were transplanted to an area with a moisture gradient. For *Stipa* spp., the slope of foliar δ^13^C and mean annual precipitation along the transect was significantly steeper than that of foliar δ^13^C and mean annual precipitation after the watering treatment. For *L. chinensis*, there was a general decreasing trend in foliar δ^13^C under the different (increasing) watering levels; however, its populations showed an irregular relationship between foliar δ^13^C and moisture origin. Therefore, support for our hypothesis was obtained from *Stipa* spp., but not from *L. chinensis*.

## Introduction

Foliar δ^13^C is a well-documented surrogate of long-term water-use-efficiency (WUE), which is determined by both photosynthesis and transpiration. Since factors that affect either of these two processes also have effects on WUE, WUE is considered an integrated measure of physiological status and environmental conditions (Farquhar et al. 1989). Extensive evidence indicates a geographic pattern, particularly at large spatial scales, of decreasing δ^13^C with increasing moisture in plants, although the explanation for this pattern varies across studies (Schulze et al. 2006; Wittmer et al. 2008; Luo et al. 2009; Diefendorf et al. 2010).

Both genetic factores and plasticity could contribute to the above-mentioned spatial pattern (Kohorn et al. 1994; Lauteri et al. 2004; Corcuera et al. 2010). Several studies have suggested the existence of a plastic response in many plants that show a decrease in δ^13^C with an increase in moisture (for example, as a result of irrigation or rainfall) (Brock and Galen 2005; Hausmann et al. 2005). In addition, there are reports on genetic determination for the observed patterns of δ^13^C increase with climatic aridity (Comstock and Ehleringer 1992). Being a quantitative trait that is subjected to continuous genetic variation, WUE (and its long-term surrogate, δ^13^C) is under the influence of both genetic and environmental factors (Chen et al. 2011). WUE, or δ^13^C, reflects the balance between the photosynthesis rate and stomatal conductance. With an increase in water availability, the reduction in the number of stomata, which are the limiting factors for photosynthesis, results in decreased levels of δ^13^C (Diefendorf et al. 2010), and this mechanism is expected to hold true across all timescales.

If the above-mentioned genetic pattern holds true, xeric-adapted populations might be able to genetically maintain a higher level of foliar δ^13^C than mesic-adapted populations, even under the same moisture conditions. However, if the plastic response holds true, lower/higher foliar δ^13^C might be observed when moisture is below/above the average. It might be speculated that, if both the genetic and plastic patterns hold true, the variation in foliar δ^13^C observed along a spatial moisture gradient would be more obvious than the variation arising purely from a plastic response to moisture change.

## Materials and Methods

### Studied species

The species studied were *Leymus chinensis* and *Stipa grandis*. *L. chinensis* has long been identified as an euryhydric species, which occupies large areas as a dominant or co-dominant species in eastern parts of the Eurasian Steppes (Siberia, Mongolia, far-east Russia, Northern China and Korea) and thrives in a diverse range of habitats (Liu et al. 2007) (The Integrated Investigation Team in Inner Mongolia and Ningxia, CAS 1985). Throughout its geographic range, *L. chinensis* coexists with *Stipa* spp., alternately dominating communities, from the drier (western) “desert steppe” to the wetter (eastern) “meadow steppe”. In contrast to *L. chinensis*, however, each species of the *Stipa* genus has a limited distribution. In the middle part of the geographic range of *L. chinensis*, the most abundant species of *Stipa* is *S. grandis*, and the communities dominated or co-dominated by *S. grandis* are often named “typical steppe” (The Integrated Investigation Team in Inner Mongolia and Ningxia, CAS 1985; CAS).

### Field investigation

**Figure 1:**
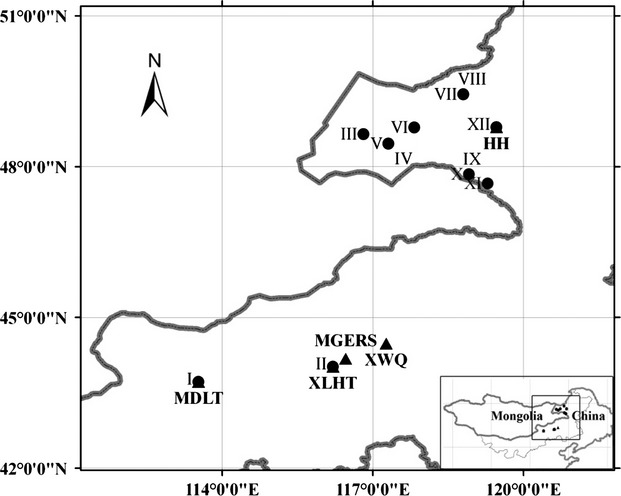
Locations of the transplantation sites along the transect in Inner Mongolia in 2009. Sites are shown as closed black circles and are numbered I to XII along the transect. The five transplantation sites are labeled as follows: Mandulatu (MDLT); Xilinhaote (XLHT); Maodeng Grassland Ecological Research Station (MGERS); Xiwuqi (XWQ); and Huihe (HH).

In 2009, 12 sites were selected from west to east along a precipitation gradient in Inner Mongolia (Fig. 1 and Table S1). At each site, a baseline was first specified (by observation) along the direction presumed to represent maximum environmental variance. Next, mature leaves of *L. chinensis* and *Stipa* spp. were collected at six points (replications) along the line. The distance between two neighboring points was 10 m. Leaves were killed by subjecting them to microwaves immediately after collection and air-dried until an oven was available.

Mean annual precipitation (MAP) at each site was established by linear interpolation of 40 weather stations distributed along the transect. MAP was computed for each station between 1950 and 2002.

### Common garden experiment

In all five sites were chosen along the transect: Mandulatu (MDLT: 43°43.212′N, 113°31.640′E); Xilinhaote (XLHT: 44°01.313′N, 116°12.433′E); Xiwuqi (XWQ: 44°28.759′N, 117°15.977′E); Huihe (HH: 48°46.792′N, 119°27.825′E), and Maodeng Grassland Ecological Research Station (MGERS: 44°10.711′N, 116°27.467′E). MDLT is located in the desert steppes; XLHT, XWQ, and MGERS in the “typical” steppes; and HH in the meadow steppes. A total of 120 ramets of *L. chinensis* were transplanted to our field experimental site at MGERS, located in the middle part of Inner Mongolia, China, from the other four sites within a maximum of 3 days. A total of 108 bunches of *S. grandis* were collected from XWQ alone, because these plants were not found at MDLT, XLHT, and HH. These bunches of *S. grandis* were immediately transplanted to our field experimental site at MGERS.

For the common garden experiment, 12 plots (2 m × 2 m) were set up, and 49 holes (diameter: 15 cm; depth: 40 cm; layout: 7 × 7 matrix) were evenly dug in each plot by using a soil corer. Ten ramets of *L. chinensis* and nine bunches of *S. grandis* from each transplantation site were randomly selected and inserted into the holes.

Up until the end of the growing season in late September 2009, all the transplanted ramets (for *L. chinensis*) or bunches (for *S. grandis*) were watered to ensure their survival. In 2010, four levels of watering treatments (0, 100, 200 and 300 mm) were randomly assigned to the 12 plots, with three replications for each level. The amount of water for each level was evenly applied 10 times during the growing season, from June 13 to September 3. No treatment was applied if natural rainfall occurred during the growing season.

After the treatment for one season, mature leaves were collected in late September 2010. The local *L. chinensis* and *S. grandis* plants growing in each plot were also harvested. *L. chinensis* samples collected from MDLT, XLHT, MGERS, XWQ, and HH were defined as L-1, L-2, L-3, L-4, and L-5, respectively. S-3 and S-4 denote the *S. grandis* samples from MGERS and XWQ.

### Carbon isotope measurement

All plant materials were dried at 65°C and ball-milled (MM200, Fa. Retsch, Haan, Germany) for δ^13^C measurement by using an IsoPrime100 Stable Isotope Ratio Mass Spectrometer (Isoprime Ltd, U.K). Stable isotope abundances are reported as:


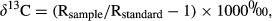


where R is the ^13^C/^12^C ratio of either the sample or the standard (Pee Dee belemnite, PDB). The standard deviation of repeated measurements of laboratory standards was ±0.15‰.

### Data analyses

All statistical analyses were performed using R 2.10.0 (R Development Core Team 2009). Correlation between foliar δ^13^C and precipitation was tested by linear regression. Differences among regression slopes were determined using Standardised Major Axis Tests & Routines (SMATR), a freely available program (Falster et al. 2006). In the field investigation, two-way analysis of variance (ANOVA) was used to test the species and sites on foliar δ^13^C values. The effects of the transplanting site (origin) and watering level (environment) on foliar δ^13^C values in the common garden experiment were analyzed using the linear model *V*_P_ = *V*_O_ + *V*_E_ + *V*_O×E_ + *V*_err_, where *V*_P_ denotes the total variance in δ^13^C; *V*_O_ is the origin variance; *V*_E_ is the environmental variance (i.e., watering-induced variance); *V*_O × E_ is the interaction between origin and environment; and *V*_err_ is the variance of residuals (Strand and Weisner 2004; Pigliucci 2005). Tukey's method was used for a multiple comparisons of the populations of *L. chinensis*.

## Results

### Foliar δ^13^C of *L. chinensis* and *Stipa* spp. along the transect

**Figure 2:**
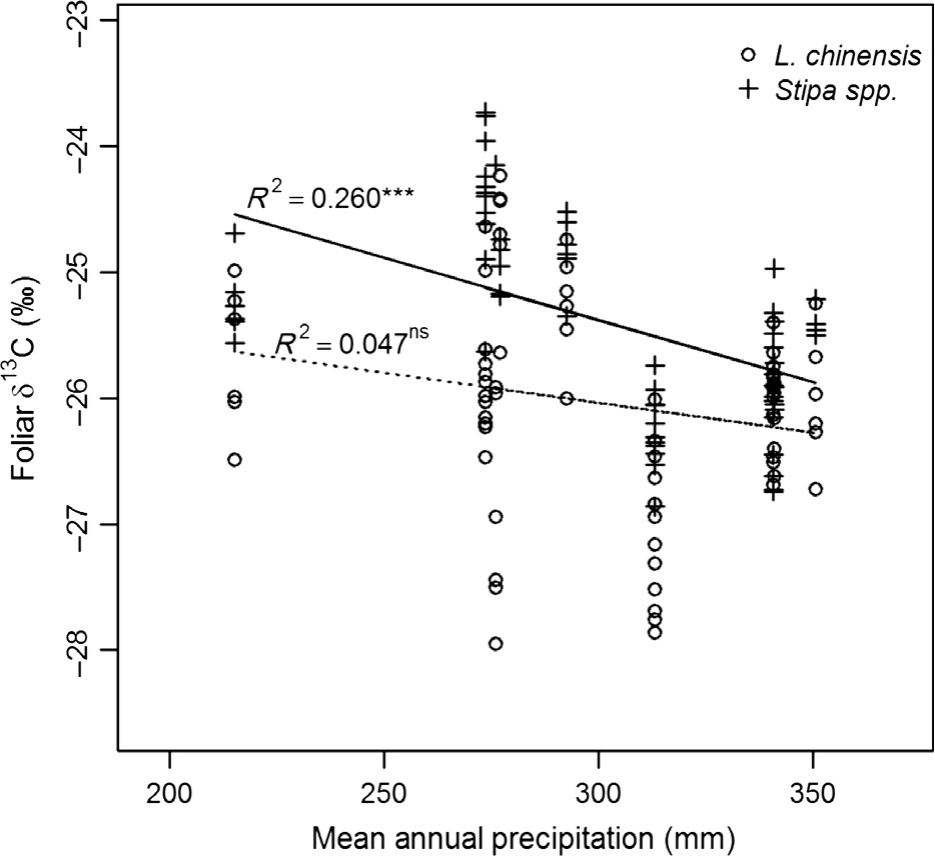
Response of foliar δ^13^C to mean annual precipitation for *Leymus chinensis* (open circles) and *Stipa* spp. (crosses) from middle to eastern Inner Mongolia, China. The three asterisks (***) denote a statistically significant regression slope at the α = 0.001 level, whereas “ns” means “not significant at the α = 0.05 level”. The dotted line represents *Stipa* spp. and the solid line one represents *L. chinensis*.

The slope of the regression between δ^13^C and MAP was extremely significant for *Stipa* spp. (*P* = 1.219E^−5^), whereas it was almost significant for *L. chinensis* (*P* = 0.068; Fig. 2). *L. chinensis* had a lower (more negative) foliar δ^13^C level than *Stipa* spp. (paired *t*-test, *P* = 0.012). The two-way ANOVA results for the transect experiment indicated an extremely significant species×site interaction (*P* = 2.998E^−7^), where *Stipa* spp. were considered to be a “species”.

### Intra-species responses of foliar δ^13^C to watering

**Figure 3:**
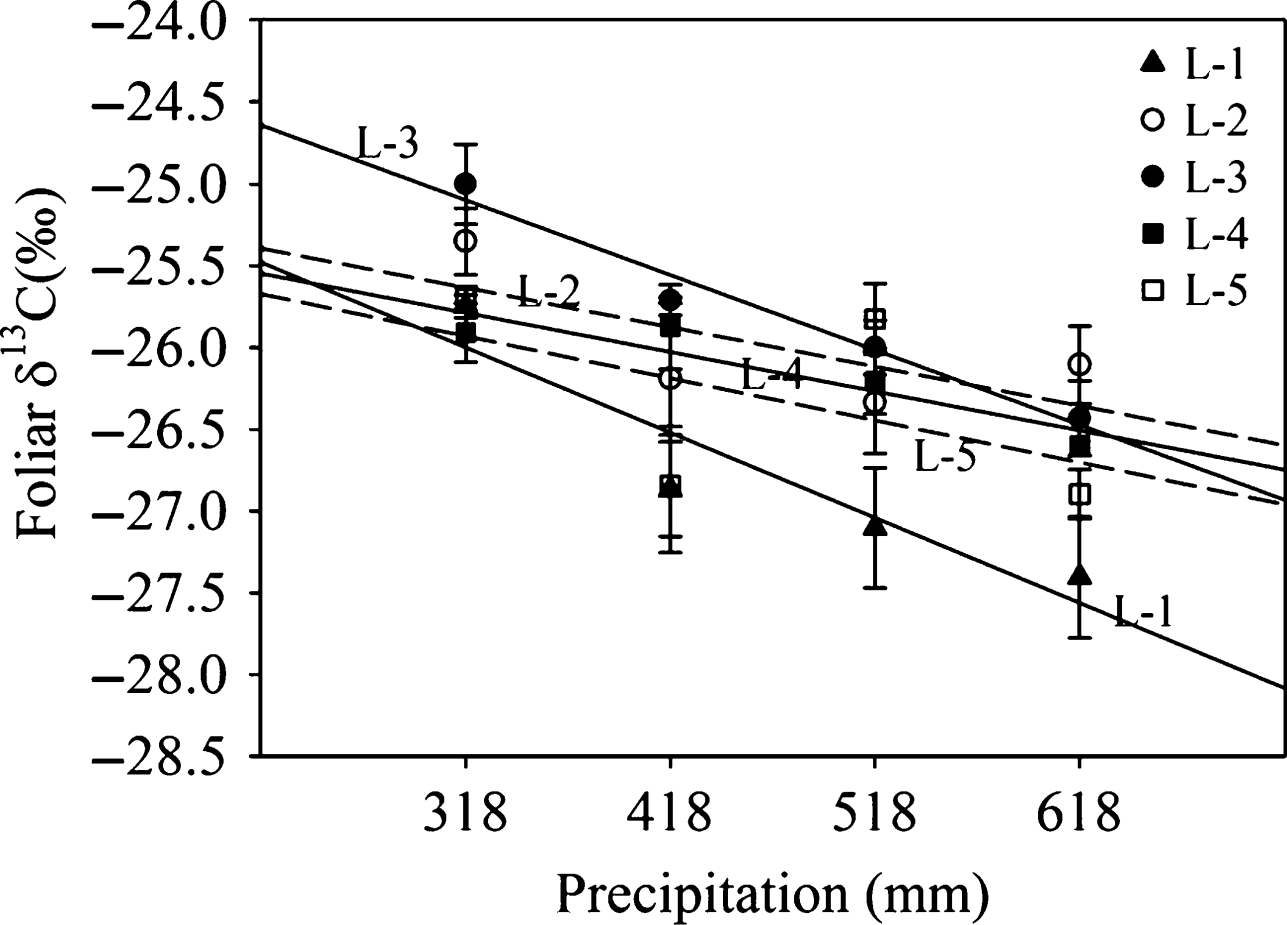
Response pattern of foliar δ^13^C to different precipitation conditions for *Leymus chinensis* from different origins. L-1, L-2, L-3, L4, and L-5 denote *L. chinensis* transplanted from MDLT, XLHT, MGERS, XWQ, and HH, respectively. Each point is the mean foliar δ^13^C ± 1SE.

As shown in Fig. 3, foliar δ^13^C level generally decreased under the different (increasing) watering levels for *L. chinensis* samples of different origins. The *P*-values of the slope tests for L-1, L-2, L-3, L-4, and L-5 were calculated as 0.005, 0.118, 0.000, 0.110 and 0.238, respectively (Fig. 3).

**Figure 4:**
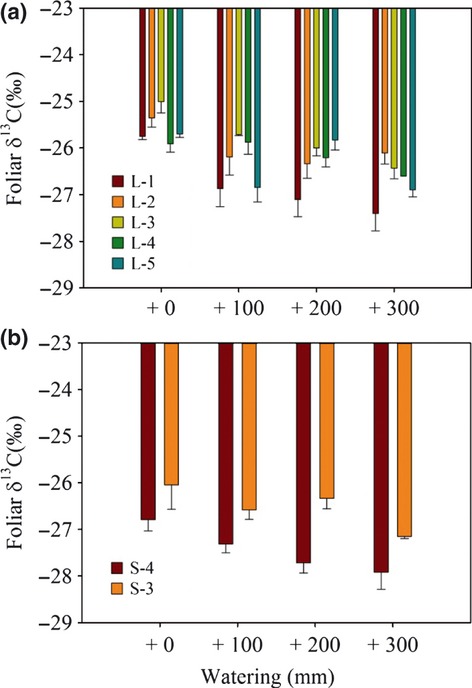
Effects of origin on foliar δ^13^C for (a) *Leymus chinensis* and (b) *Stipa grandis* along the moisture gradient. L-1, L-2, L-3, L4, and L-5 denote *L. chinensis* transplanted from MDLT, XLHT, MGERS, XWQ, and HH, respectively, with the number increasing relative to a decrease in aridity of the habitat. S-3 and S-4 denote *S. grandis* transplanted from the same habitats like L-3 and L-4, respectively. Error bars are ±1SE.

Since origin was found to be a significant factor according to the two-way ANOVA results for both the species (*L. chinensis*, *P* = 0.012; *S. grandis*, *P* = 0.011), multiple comparisons were conducted using Tukey's method. L-1, which originated from the desert steppes, had significantly lower foliar δ^13^C level than those in L-2, L-3 and L-4. Although the *P*-value was lower, the difference in foliar δ^13^C level between L-1 and L-5 was not statistically significant (*P* = 0.113). Despite origin having a significant effect on foliar δ^13^C in *L. chinensis*, the effect was not linear. L-1 had the lowest foliar δ^13^C value in three out of the four watering levels (Fig. 4a).

It is worth noting that origin had a consistent effect across species, if only L-3, L-4, S-3 and S-4 were considered. The habitat of S-3 was drier than that of S-4, and δ^13^C values for S-3 were higher than those for S-4 under all watering levels (Fig. 4b). Similar results were noted for *L. chinensis* species, L-3 and L-4 (Fig. 4a).

### Comparison of the response pattern between the transect and watering experiments

**Figure 5:**
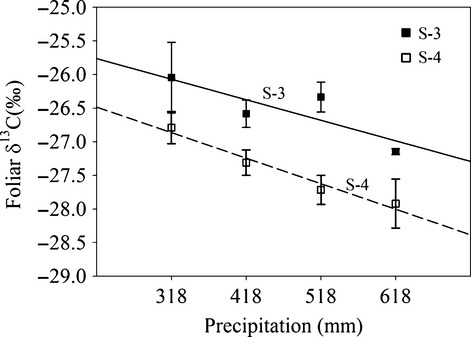
Response pattern of foliar δ^13^C to the different precipitations for *Stipa grandis* from different origins. S-3 and S-4 denote *S. grandis* transplanted from MGERS and XWQ, respectively. Each point is the mean foliar δ^13^C ± 1SE.

The *P*-values of the slope tests for watering ewperiments on S-3 and S-4 were not significant (*P* = 0.049 and 0.005, respectively) and hence they were combined to form one slope for *S. grandis*. The combined slope was calculated as −0.006952‰·mm^−1^, which differs significantly from zero (*P* = 0.008). The slope between δ^13^C and MAP for *Stipa* spp. in the transect experiment was −0.019319‰·mm^−1^, which also differed significantly from zero (*P* = 0.000, Fig. 2). The two slopes, the combined slope obtained from the watering experiment and the other slope based on the transect sampling, differed significantly (*P* = 0.001; Fig. 5).

## Discussion

*Stipa* spp. is generally more conservative than *L. chinensis* across the transect along which they coexist (Fig. 2). The combined slope of foliar δ^13^C and MAP for *Stipa* spp. along the transect (−0.0193) was very close to the “universal” slope for all C3 species reported by Prentice et al. (2011): −0.014. After converting δ^13^C to ^13^Δ, the combined slope for *Stipa* spp. (0.0193) was also close to the value of 0.0100‰/mm, or 0.0105 (^13^Δ/MAP), as reported by Wittmer et al. (2008). The slightly greater value of our slope might be attributed to the selection of the sampling area in our study that extended further toward the east, including the wet-adapted *S. baicalensis*.

The slope of foliar δ^13^C and MAP for *Stipa* spp. across the transect represents the effects of both genetic and phenotypic plasticity (Fig. 2), whereas the slope of δ^13^C and watering in the common garden experiment represents only the plastic response (Fig. 5), the former is significantly steeper than the latter. A plausible explanation for this is that, in addition to the plasticity pattern that has arisen (as seen in Fig. 5), there was a genetically determined pattern of foliar δ^13^C. Similar to other species reported in previous research (Comstock and Ehleringer 1992; Zhang et al. 1993; Dudley 1996), *Stipa* populations have a tendency to show increased foliar δ^13^C with aridity of their original environment, even under common conditions. Since under natural conditions, xeric-adapted populations are generally exposed to less moisture than mesic-adapted populations, a plastic response would further increase the foliar δ^13^C levels.

On the other hand, the genetic and plastic patterns differed in *L. chinensis*. The plasticity response pattern showed a linearly negative relationship between foliar δ^13^C and water availability (Fig. 3), which was similar to that noted in *S. grandis* (Fig. 5). However, the genetically determined response pattern was unique to *L. chinensis*. Populations derived from a moderately arid environment (sites with average MAP) were thought to show the highest foliar δ^13^C, whereas those (L-1) derived from the dryer end of the transect (site with the lowest MAP) maintained consistently lower δ^13^C values (Fig. 4). Although a significant relationship between δ^13^C and moisture availability was observed in *L. chinensis*, the particular (irregular) pattern in intrinsic δ^13^C among populations reduced the significance of the spatial relationship between δ^13^C and MAP (Fig. 2).

The nonlinear response pattern underlying the genetic variation of *L. chinensis* could explain the lack of agreement among previously published results. Chen et al. (2002) reported a negative relationship between δ^13^C and soil moisture for *L. chinensis*, this assessement was performed in plants sampled along an environmental gradient in the Xilin river basin. A similar relationship between δ^13^C and MAP for *L. chinensis* was observed by Su et al. (2000) along the North-East China Transect (NECT). On the basis of the results obtained from 171 species sampled along the NECT, Prentice et al. (2011) proposed a universal scaling relationship between δ^13^C and aridity, which persists both within and across species. Although most species in their study followed this universal pattern, *L. chinensis* was one of the two exceptions that showed a reverse relationship to that shown in Fig. 8 in Prentice et al. (2011). This exception could be explained by the unique genetic pattern of *L. chinensis*. Su et al. (2000) and Chen et al. (2002) sampled *L. chinensis* from the middle to eastern region of the NECT, while Prentice et al. (2011) sampled this species from the western region of the NECT.

In conclusion, in spite of the evidence for a genetic response pattern unique to *L. chinensis*, the underlying mechanism remains unknown and warrants further research.

## Supporting Information

**Table S1.** Geographical features of the sampling sites in Inner-Mongolia
