# Supplementary material for: Foliar δ13C response patterns along a moisture gradient arising from genetic variation and phenotypic plasticity in grassland species of Inner Mongolia
Source: Ecol Evol. 2012 Dec 27;3(2):262–7. doi: 10.1002/ece3.453 (PMC3586636; doi:10.1002/ece3.453)
Supplement: Supplementary file 1 [file ece30003-0262-SD1.doc]

**Table S1. Geographical features of the sampling sites in Inner-Mongolia**

| Plot Number | Location | | Altitude (m)  (m) | Annual Precipitation (mm) | Species Sampled |
| --- | --- | --- | --- | --- | --- |
| I | N43°43.212′ | E113°31.640′ | 1027 | 178.5401 | *L. chinensis*  *Stipa* spp. |
| II | N44°01.313′ | E116°12.433′ | 1051 | 241.0401 | *L. chinensis*  *Stipa* spp. |
| III | N48°38.593′ | E116°48.935′ | 553 | 225.2809 | *L. chinensis*  *Stipa* spp. |
| IV | N48°27.197′ | E117°18.799′ | 624 | 236.5494 | *L. chinensis*  *Stipa* spp. |
| V | N48°27.197′ | E117°18.799′ | 624 | 236.5494 | *L. chinensis*  *Stipa* spp. |
| VI | N48°46.457′ | E117°49.671′ | 550 | 257.9259 | *L. chinensis*  *Stipa* spp. |
| VII | N49°25.959′ | E118°48.209′ | 616 | 289.2407 | *L. chinensis*  *Stipa* spp. |
| VIII | N49°25.959′ | E118°48.209′ | 616 | 289.2407 | *L. chinensis*  *Stipa* spp. |
| IX | N47°50.541′ | E118°54.990′ | 757 | 277.2253 | *L. chinensis*  *Stipa* spp. |
| X | N47°50.541′ | E118°54.990′ | 757 | 277.2253 | *L. chinensis*  *Stipa* spp. |
| XI | N47°39.521′ | E119°17.437′ | 871 | 326.9197 | *L. chinensis*  *Stipa* spp. |
| XII | N48°46.792′ | E119°27.825′ | 680 | 299.2222 | *L. chinensis*  *Stipa* spp. |
